# The Pathogenic Neisseria Use a Streamlined Set of Peptidoglycan Degradation Proteins for Peptidoglycan Remodeling, Recycling, and Toxic Fragment Release

**DOI:** 10.3389/fmicb.2019.00073

**Published:** 2019-01-31

**Authors:** Ryan E. Schaub, Joseph P. Dillard

**Affiliations:** Department of Medical Microbiology and Immunology, School of Medicine and Public Health, University of Wisconsin-Madison, Madison, WI, United States

**Keywords:** peptidoglycan (PG), Neisseria, peptidoglycan (PG) hydrolases, lytic transglycosylase, *O*-acetylation, lipoproteins, NOD1

## Abstract

*Neisseria gonorrhoeae* and *Neisseria meningitidis* release peptidoglycan (PG) fragments from the cell as the bacteria grow. For *N. gonorrhoeae* these PG fragments are known to cause damage to human Fallopian tube tissue in organ culture that mimics the damage seen in patients with pelvic inflammatory disease. *N. meningitidis* also releases pro-inflammatory PG fragments, but in smaller amounts than those from *N. gonorrhoeae*. It is not yet known if PG fragment release contributes to the highly inflammatory conditions of meningitis and meningococcemia caused by *N. meningitidis*. Examination of the mechanisms of PG degradation and recycling identified proteins required for these processes. In comparison to the model organism *E. coli*, the pathogenic Neisseria have far fewer PG degradation proteins, and some of these proteins show differences in subcellular localization compared to their *E. coli* homologs. In particular, some *N. gonorrhoeae* PG degradation proteins were demonstrated to be in the outer membrane while their homologs in *E. coli* were found free in the periplasm or in the cytoplasm. The localization of two of these proteins was demonstrated to affect PG fragment release. Another major factor for PG fragment release is the allele of *ampG*. Gonococcal AmpG was found to be slightly defective compared to related PG fragment permeases, thus leading to increased release of PG. A number of additional PG-related factors affect other virulence functions in Neisseria. Endopeptidases and carboxypeptidases were found to be required for type IV pilus production and resistance to hydrogen peroxide. Also, deacetylation of PG was required for virulence of *N. meningitidis* as well as normal cell size. Overall, we describe the processes involved in PG degradation and recycling and how certain characteristics of these proteins influence the interactions of these pathogens with their host.

## Peptidoglycan (Pg) Structure in Neisseria

**FIGURE 1:**
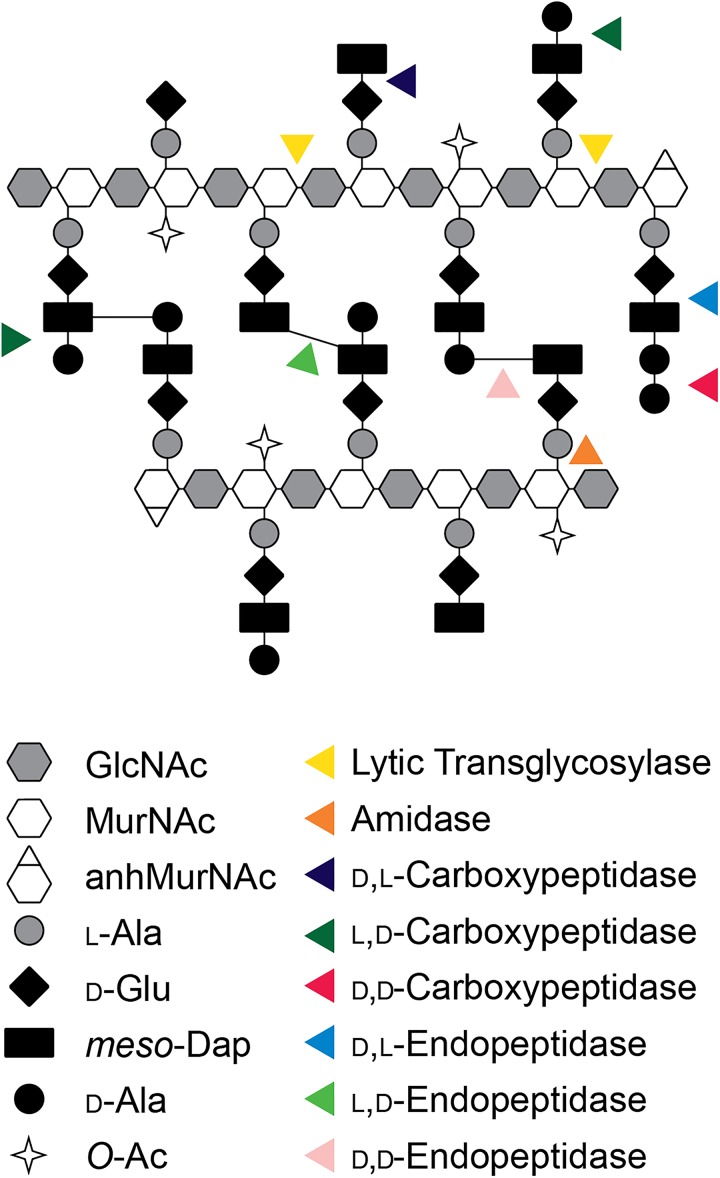
Model of the PG structure of Neisseria and cleavage sites of by different classes of PGases. GlcNAc, *N*-acetylglucosamine; MurNAc, *N-*acetylmuramic acid; anhMurNAc, 1,6-anhydro-*N*-acetylmuramic acid; L-Ala, L-alanine; D-Glu, D-glutamic acid; *meso-*Dap, *meso*-diaminopimelic acid; D-Ala, D-alanine; *O*-Ac, *O-*acetylation.

The pathogenic Neisseria attracted the attention of peptidoglycan (PG) researchers due to the propensity of these bacteria to release small PG fragments during growth (Rosenthal, 1979; Sinha and Rosenthal, 1980). During gonococcal infections, these released PG fragments induce an inflammatory response in the human host that causes tissue damage in the Fallopian tubes and may exacerbate the pathology of urethral, uterine, and disseminated infections. However, the structure of Neisseria PG is not unusual. In *N. gonorrhoeae* and *N. meningitidis*, known as gonococci (GC) and meningococci (MC), the PG composition is highly similar to that seen in *Escherichia coli* and many other Gram-negative bacterial species (Dougherty, 1985; Glauner et al., 1988; Antignac et al., 2003b). The glycan strands are composed of repeating β-(1,4)-linked disaccharides of GlcNAc-β-(1,4)-MurNAc. Peptides are attached to the MurNAc and consist of two to five amino acids of the sequence L-Ala-D-Glu-*meso*-Dap-D-Ala-D-Ala (Figure 1). In *N. gonorrhoeae*, approximately 40% of the peptides are crosslinked to peptides on adjacent PG strands to form the cell wall, although there is some variation in the degree of crosslinking between strains (Rosenthal et al., 1980). The crosslinks are formed between the fourth amino acid D-Ala on one strand and the third amino acid *meso*-Dap on the other for a majority of the crosslinks. Crosslinks are also formed between *meso*-Dap on one strand and *meso*-Dap on the other strand. It was reported that *N. meningitidis* cell wall does not contain Dap–Dap crosslinks (Antignac et al., 2003b), but more recent experiments indicate that Dap–Dap crosslinks occur in both *N. gonorrhoeae* and *N. meningitidis* (Woodhams, 2013). The peptide chains in the intact cell wall are mostly tetrapeptides (75%) or tripeptides (25%), with dipeptides and pentapeptides making up a small fraction (Dougherty, 1985). There are two significant differences between Neisseria PG and that of *E. coli*. Neisseria have *O*-acetylation at the C6-hydroxyl on about 50% of the MurNAc residues (Rosenthal et al., 1982). This modification controls the function of lytic transglycosylases (LTs) and serves to limit PG degradation by host lysozyme (Blundell et al., 1980; Rosenthal et al., 1982). The second difference is that *E. coli* has proteins covalently attached to the PG such as Braun’s lipoprotein (Lpp), whereas GC and MC do not have proteins covalently attached to PG (Wolf-Watz et al., 1975).

## Release of Peptidoglycan Fragments Into the Milieu

**FIGURE 2:**
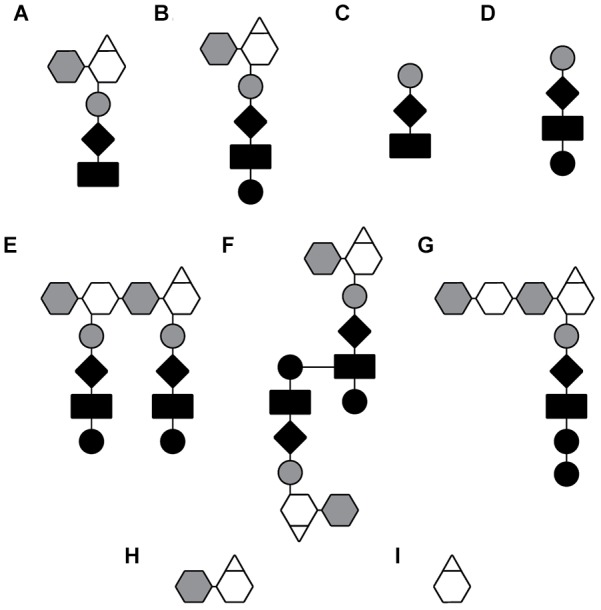
Depiction of PG fragments released by GC and MC. PG fragments listed in Table 1. **(A)** Anhydro-tripeptide monomer, **(B)** anhydro-tetrapeptide monomer, **(C)** free tripeptide, **(D)** free tetra peptide, **(E)** glycosidically linked dimer, **(F)** peptide-linked dimer, **(G)** tetrasaccharide peptide, **(H)** free anhydro-disaccharide, and **(I)** anhydro-MurNAc.

Raoul S. (Randy) Rosenthal found that *N. gonorrhoeae* had a high rate of PG turnover, and for over a decade he worked to characterize the PG fragments released and their effects on infection. The most abundant PG fragments released from GC are the PG monomers (GlcNAc-anhMurNAc-tripeptide and GlcNAc-anhMurNAc tetrapeptide) and the free peptides (Ala–Glu–Dap and Ala–Glu–Dap–Ala). The free tripeptide and the tripeptide monomer are agonists for the pattern-recognition receptor NOD1 in humans, and these molecules likely contribute to the large inflammatory responses seen in a variety of *N. gonorrhoeae* infections (Girardin et al., 2003a). In the *ex vivo* model of gonococcal pelvic inflammatory disease, PG fragments from GC were shown to be sufficient to cause death and sloughing of ciliated cells in human Fallopian tubes and to recapitulate the tissue damage seen in patients with pelvic inflammatory disease (Melly et al., 1984). In addition to the release of PG monomers and free peptides, *N. gonorrhoeae* and *N. meningitidis* release a variety of other PG fragments. These include both glycosidically linked and peptide-linked PG dimers, a tetrasaccharide with a single attached peptide, free disaccharide, and anhydroMurNAc (Rosenthal, 1979; Sinha and Rosenthal, 1980; Woodhams et al., 2013) (see Table 1 and Figure 2).

**Table 1:** PG degradation enzymes required for release of specific PG fragments.

	PG fragment	AKA	Required enzymes	Significance
A	G-aM-3	Anhydro-tripeptide monomer	LtgA or LtgD, PBP3 or PBP4, LdcA	Toxic to FTOC, NOD1 agonist
B	G-aM-4	Anhydro-tetrapeptide monomer	LtgA or LtgD	Mouse NOD1 agonist, TCT
C	L-Ala-D-Glu-mDAP	Free tripeptide	AmiC, PBP3 or PBP4, LdcA	NOD1 agonist
D	L-Ala-D-Glu-mDAP-D-Ala	Free tetrapeptide	AmiC, PBP3 or PBP4	Mouse NOD1 agonist
E	G-M(4)-G-aM(4)	Glycosidic dimer	LTs	Converted by host lysozyme to NOD2 agonist
F	G-aM-4-4-aM-G	Peptide-linked dimer	LTs	
G	G-M-G-aM(4)	Tetrasaccharide-peptide	AmiC, LtgC	
H	G-aM	Free disaccharide	AmiC, LtgC	
I	aM	Anhydro-MurNAc	NagZ, AmiC	

*G, N-acetylglucosamine; M, N-acetyl muramic acid; aM, 1,6-anhydro-N-acetyl muramic acid; 3, tripeptide; 4, tetrapeptide. Ltg indicates an unspecified lytic transglycosylase. FTOC, human fallopian tube tissue in organ culture.*

Neisseria are not the only bacterial species that release PG fragments during growth. Work from Bill Goldman et al. (1982) over many years described the release of the PG monomer GlcNAc-anhMurNAc tetrapeptide by *Bordetella pertussis* (Rosenthal et al., 1987). Known as tracheal cytotoxin or TCT, the PG monomer was found to lead to loss of ciliated cells in hamster tracheal organ culture and production of inflammatory cytokines and nitric oxide by primary tracheal cells from that tissue (Heiss et al., 1993). In *E. coli*, PG fragments are released, but in much smaller amounts than in Neisseria or Bordetella. *E. coli* release 3–8% of their glycan-containing PG fragments generated during growth. The fragments that are released are the smaller, more broken down pieces of PG and consist of GlcNAc-anhMurNAc and free peptides (Goodell and Schwarz, 1985; Park and Uehara, 2008). *Vibrio fischeri* was shown to release PG monomers, and these PG fragments serve to stimulate tissue remodeling in the Hawaiian bobtail squid (*Euprymna scolopes*) that is in a mutualistic relationship with the bacteria (Koropatnick et al., 2004). It is interesting to note that in *V. fischeri* symbiosis, the PG fragments cause the loss of ciliated cell appendages, which is reminiscent of the loss of ciliated cells in the *B. pertussis* and *N. gonorrhoeae* infections. A number of other bacterial species stimulate a NOD1 response during bacterial infections, which suggests that those species also release PG fragments. *Shigella flexneri, Helicobacter pylori*, and *Chlamydia trachomatis* among other species, have been shown to stimulate NOD1 responses, suggesting that those bacteria release PG fragments (Viala et al., 2004; Welter-Stahl et al., 2006; Nigro et al., 2008).

## Neisseria Have a Small Set of Peptidoglycanases

Peptidoglycan degrading enzymes, broadly called peptidoglycanases (PGases), continuously act to modify and degrade PG. Some of these PGases act to modify PG by trimming peptide stems in ways that affect crosslinking and maturation. Others act to cleave PG to allow for the insertion of new glycan strands and to allow dividing cells to separate. GC and MC have a reduced set of PGases compared to most other Gram-negative bacteria. The reduction extends over many different classes of PGases that include LTs, endopeptidases/carboxypeptidases (EPs/), *N*-acetylmuramoyl-L-alanine amidases (amidases), and other activator or accessory proteins. With fewer PGases there is less redundancy of PG degradation functions. A consequence of having a simplified set of PGases is that single gene mutations can present phenotypic differences.

A prime example of the simplified nature of Neisseria PGases is the amidases. The amidases are responsible for removing the peptide stem from the glycan backbone. *E. coli* and many other Gram-negatives have four periplasmic amidases. In *E. coli* three amidases, AmiA, AmiB, and AmiC, are cell separation amidases. These three amidases have two activators. EnvC activates AmiA and AmiB, and NlpD activates AmiC (Uehara et al., 2010). The inactivation of one or two amidases has little or no effect of cell separation. However, if genes for all three cell separation amidases are mutated then the cells form long chains of unseparated cells (Priyadarshini et al., 2006). In contrast, *N*. *gonorrhoeae* has only a single cell separation amidase, AmiC. A single mutation of either *amiC*, or *nlpD*, which encodes the activator protein, results in a separation phenotype that causes cells to form clumps sharing cell walls (Garcia and Dillard, 2006).

A similar reduction has been observed for LTs, the enzymes that cleave the MurNAc-β-(1,4)-GlcNAc bond of the glycan backbone. As of a current count, the core genome of Neisseria includes at seven putative LTs, while the *E. coli* genome encodes nine LTs (Dik et al., 2017). Single LT mutant phenotypes have been observed for the many LTs in GC, but not in *E. coli* (Lommatzsch et al., 1997). Mutation of *ltgC* in GC, or its homolog *gna33* in MC, causes cell separation defects similar to what is observed in an *amiC* mutant (Adu-Bobie et al., 2004; Cloud and Dillard, 2004). The same is not true of the *E. coli* homolog MltA. Mutation of *mltA* alone or in combination of two other LT genes, *slt* and *mltB*, showed no phenotype (Lommatzsch et al., 1997).

Neisseria have two LTs responsible for the production of PG monomers. These LTs, LtgA and LtgD, are homologs of Slt70 and MltB in *E. coli*. LtgA and LtgD are responsible for creating nearly all of the tripeptide and tetrapeptide PG monomers released by GC (Cloud-Hansen et al., 2008). Mutation of *ltgA* results in a 38% decrease in PG monomers while mutation of *ltgD* results in 62% decrease (Cloud and Dillard, 2002). Mutation of both *ltgA* and *ltgD* results in the absence of released of PG monomers including the NOD1 agonist, tripeptide monomer (Cloud-Hansen et al., 2008).

Other single gene mutation phenotypes include increased release of peptide-linked PG dimers and a reduction in tripeptide monomers observed when deleting the L,D-carboxypeptidase gene, *ldcA* (Lenz et al., 2017). Deletion of the D,D-carboxy/endopeptidase gene *dacB* causes the accumulation of pentapeptide in the sacculi and the release of more pentapeptide monomer (Obergfell et al., 2018). Similar results were seen for an *ldcA* mutant in MC and are expected to occur in the MC *dacB* mutant (Woodhams, 2013; Lenz et al., 2017).

Gonococci appears to be missing a number of Class C PBPs known to be peptidases. A class of strict D,D-carboxypeptidases, called Type-5 PBPs, is present in many Gram-negatives. *E. coli* has three members of this family of proteins encoded by *dacA, dacC*, and *dacD* (Sauvage et al., 2008). Of these genes, only *dacC* is present in Neisseria, but the conserved active site residues are missing (Obergfell et al., 2018). AmpH-like PBPs are also absent in the pathogenic Neisseria (GenBank: AE004969.1) (Benson et al., 2018).

Peptidoglycan synthesis proteins are reduced in the GC and MC in a similar trend as PG degrading proteins. GC has only one Class A bifunctional transglycosylase/transpeptidase PBP and one Class B transpeptidase PBP in contrast to *E. coli* that has three Class A PBPs and two Class B (Sauvage et al., 2008). This difference in high molecular mass (HMM) PBPs could be due to the absence of elongation-related proteins in coccoid bacteria. A similar reduction of HMM PBPs is also observed in coccoid Gram-positive bacteria. *Bacillus subtilis* has four Class A PBPs and six Class B PBPs while *Staphylococcus aureus* has only one Class A PBP, and three Class B PBPs. The reduced number of PG synthesis genes in Neisseria and other coccoid bacteria that do not undergo elongation suggests that the genes necessary for a cell elongation complex are absent in these bacteria.

## Pgase Localization in Neisseria

In addition to a reduction of PGases some of the PGases in Neisseria have unique subcellular localization compared to other species. This is especially true of the LTs with four of seven core LTs predicted to have localizations different than what has been observed in *E. coli* (Table 2). The cellular localization of periplasmic proteins is determined by the signal sequence. Lipoproteins have a signal sequence that contains a “lipobox” consisting of the protein sequence LxxC where x represents a small amino acid (Kovacs-Simon et al., 2011). The cysteine of the lipobox is lipidated, and then the protein is either transferred to the outer membrane or retained in the inner membrane depending of the amino acid following the cysteine by what is known as the +2 rule (Seydel et al., 1999).

**Table 2:** Subcellular localization of LTs and peptidases in GC and *E. coli.*

Type	GC protein	Locus	SignalP^a^	LipoP^b^	EC protein	SignalP^a^	LipoP^b^
LTs	LtgA	NGO_2135	Y	SpII+2=S	Slt70	Y	SpI
	LtgB	NGO_1033	Y	SpI	MltC	Y	SpII+2=S
	LtgC	NGO_2048	Y	SpII+2=Q	MltA	Y	SpII+2=S
	LtgD	NGO_0626	Y	SpII+2=T	MltB	Y	SpII+2=S
	LtgE	NGO_0608	Y	SpI	MltD	Y	SpII+2=Q
	LtgG	NGO_0238	Y	SpI	MltG	Y	SpI
	RlpA	NGO_1728	Y	SpI	RlpA	Y	SpII+2=T
GGI LTs	AtlA	NGrG_00979^c^	N	Cyt	Lambda R^d^	N	Cyt
	LtgX	NGrG_01000^c^	Y	Cyt	F orf169^e^	Y	SpI
Peptidases	PBB3	NGO_0107	Y	SpI	PBP4	Y	SpI
	PBP4	NGO_0327	Y	SpI	PBP7	Y	SpI
	DacC	NGO_0443	Y	SpI	PBP6	Y	SpI
	LdcA	NGO_1274	Y	SpII+2=G	LdcA	N	CYT

*^a^Prediction of a signal peptide using SignalP 3.0. Y, periplasmic localization; N, cytoplasmic localization. ^b^Prediction of subcellular localization and lipidation by LipoP. Cyt, cytoplasm; SpI, periplasmic; SpII, lipidated protein with +2 amino acid influencing inner membrane or outer membrane localization. ^c^Locus from GC strain MS11 due to the reference strain FA 1090 not possessing a GGI. ^d^E. coli lambda phage LT. ^e^LT from E. coli F-plasmid.*

LtgD is the LT responsible for creating the majority of PG monomers that are released from GC (Schaub et al., 2016). LtgD has been classified as a Family 3A LT (Dik et al., 2017). One characteristic of this family of LTs is that they are present as both membrane-bound and soluble proteins. In *E. coli*, membrane-bound lytic transglycosylase B (MltB) is anchored to the outer membrane by a lipidated cysteine residue (Ehlert et al., 1995). Proteolytic cleavage of MltB results in a soluble derivative called Slt35. *Pseudomonas aeruginosa* also has multiple Family 3A LTs (Dik et al., 2017). In this case there is no cleavage of the protein to create two forms of the protein, but there are two paralogs, one that gets anchored to the membrane and a more efficient form that is soluble (Blackburn and Clarke, 2002). In GC there is only one form of LtgD, and it is always anchored to the outer membrane (Schaub et al., 2016). If a mutation is made that results in the absence of the anchoring cysteine of LtgD to the outer membrane, then there is a decrease in the amount of PG monomers released from GC (Schaub et al., 2016). The purpose of having multiple localizations for Family 3A LTs is still unclear, but it appears that membrane localization of LtgD favors the release of PG fragments.

The localization of LtgA to the outer membrane is different in Neisseria and related species than it is in most other bacteria such as the periplasmically localized homolog in *E. coli* known as Slt70 (for soluble LT 70 kDa). The septally localized LtgA produces the majority of monomers produced in the cell with most monomers being taken up by the cytoplasmic membrane permease AmpG to be recycled. Conversely, LtgD is distributed throughout the cell and the majority of monomers produced by LtgD are released from the cell. When the lipobox cysteine of LtgA was mutated there was no change in the abundance of released monomer (Schaub et al., 2016). This result either means that outer membrane localization is not important for LtgA’s function in PG fragment release or that removal of the lipid is not a sufficient change to prevent LtgA association with the outer membrane.

Other LTs are localized differently in Neisseria. In *E. coli* MltC is a lipoprotein that localizes to the inner leaflet of the outer membrane. The Neisseria MltC homolog, LtgB, does not have a lipobox motif and is predicted to be an unanchored periplasmic protein. LtgB also lacks an N-terminal DUF3393 domain usually associated with Family 1B LTs (Dik et al., 2017). Single mutants do not have a known phenotype in either GC or *E. coli*, but the true function of these LTs may be masked by a redundant LT.

Bioinformatics indicates that other LT localizations are also different in Neisseria. The Family 1D LT, LtgE, is predicted to be a periplasmic protein unlike its *E. coli* homolog, MltD, that is predicted to be anchored to the outer membrane (Bateman and Bycroft, 2000) (Table 2). It is possible that LtgE in fact an inner membrane protein. It has a lipobox (LSVCP) with the proline at the +2 position predicting that the protein would be retained in the inner membrane (Seydel et al., 1999). This prediction is similar to what was found for the *Pseudomonas aeruginosa* LtgE homolog that is also a lipoprotein retained in the inner membrane (Lewenza et al., 2008). The SPOR-domain-containing LT named RlpA (for rare lipoprotein A) is a lipoprotein in *E. coli* as the name suggests. In GC and MC RlpA does not contain a cysteine and is not predicted to be lipidated (GenBank: AAW90349.1).

The localization of another PGase functions to create more tripeptide monomer, as mentioned above. In other bacteria LdcA is a cytoplasmic protein that functions in recycling to remove the D-Ala at the fourth position of the peptide stem (Templin et al., 1999). It is this removal of the D-Ala that allows this tripeptide to be reused. Mpl adds the tripeptide to UDP-MurNAc and then MurF then adds D-Ala–D-Ala to UDP-MurNAc tripeptide to form the PG precursor UDP-MurNAc-pentapeptide (Johnson et al., 2013). It is presumed that LdcA has the same cytoplasmic function in Neisseria. Lenz et al. (2017) found that LdcA is also present as an outer-membrane lipoprotein in GC in addition to being present in the cytoplasm. LdcA was found to be functional in the periplasm since an *ldcA* signal sequence deletion, that keeps LdcA in the cytoplasm, has the same effect on sacculi composition and PG fragment release as an *ldcA* deletion. Mutation of *ldcA* eliminated the presence of tripeptides in the sacculi while increasing the abundance of tetrapeptides. The mutation of *ldcA* altered the ratio of released PG fragments from 3:1 tripeptide to tetrapeptide monomers to nearly only tetrapeptide monomers. The *ldcA* mutants released significantly more peptide-linked PG dimer, suggesting that LdcA also cuts the L,D-Dap–Dap crosslinks. The localization of LdcA to the periplasm is necessary for the observed phenotypes (Lenz et al., 2017). Consequently, the unusual periplasmic localization of LdcA is necessary for the production of NOD1 agonists.

Another way that Neisseria cell wall proteins localize differently is within protein complexes. Recently the cell division interactome, or divisome, of *N. gonorrhoeae* has been investigated and was found to have two unique interactions and a number of interactions missing (Zou et al., 2017). An instance where interactions are missing is with the cell division transpeptidase PBP2 encoded by the gene *penA.* The *E. coli* homolog, known as PBP3 or FtsI, has been found to interact with seven different proteins during cell division. Intriguingly, gonococcal PBP2 was only found to interact with one cell separation protein, FtsW. PBP2 is the essential target of many β-lactam antibiotics. Consequently, *penA* alleles conferring β-lactam resistance are becoming common in clinical isolates. Up to 60 amino acid changes have been observed in *penA* mutants (Tomberg et al., 2017). The mutations resulting in antibiotic resistance have been found to decrease the affinity of PBP2 to β-lactams and result in less transpeptidation that causes an increase in pentapeptide stems in the sacculi of resistant strains in GC and MC (Garcia-Bustos and Dougherty, 1987; Antignac et al., 2003a). Some alleles, such as *penA41*, confer a 300-fold increase in the minimum inhibitory concentration (MIC) for ceftriaxone (Tomberg et al., 2013). It is possible that the absence of many of the PBP2 interactions observed in other organisms has allowed the incorporation of beneficial *penA* mutations that would otherwise not be viable.

## Machine-Related Pgases

One of the main functions of PG is to provide act as a barrier to macromolecules. The mesh-like structure of PG has been shown to exclude proteins larger than 50 kDa (Demchick and Koch, 1996). Larger proteins and protein complexes need to modify the PG to get past the barrier in a way that does not compromise the integrity of the PG and the cell. Several PGases only act on specific localized substrates and are often associated with larger protein complexes. One of these complexes is the Type IV pilus (Tfp). This organelle is essential for infection and is used by Neisseria for attachment to epithelial cells, twitching motility, resistance to oxidative killing, and for DNA uptake (Stohl et al., 2013; Kolappan et al., 2016). It is composed of sub-complexes that allow for the Tfp to extend through the periplasm and extend and retract the pili fibers with molecular motors. The Tfp can extend micrometers beyond the surface of the cell, and each motor has a force exceeding 100 pN (Maier et al., 2002).

An M23B zinc metalloprotease known as NGO1686 or Mpg (for metalloprotease active against PG) was originally found in a screen for genes upregulated during oxidative stress (Stohl et al., 2005, 2012). The connection of a PGase to oxidative stress was originally unclear. Later observations from Dillard and Seifert (2001) found that *mpg* mutants had altered colony morphology and led to an investigation of the Tfp. The *mpg* mutants were found to be defective in piliation, having only about one fifth as many pili as the wild-type, and the mutant exhibited low-piliation phenotypes such as lower levels of natural transformation (Stohl et al., 2013). Further investigation revealed that only functioning pili protected from oxidative stress. A series of pili mutations led to the hypothesis that *mpg* mutants do not have wild-type pili anti-retraction properties, and that Mpg acts to remodel PG to allow for the formation of an anti-retraction complex (Stohl et al., 2013).

Other PGases are also necessary for the assembly of the multi-protein Type IV pili complex (Tfp). Other PGases that influence pili formation are the low-molecular-mass penicillin-binding proteins PBP3 (aka DacB) and DacC, homologs of *E. coli* PBP4 and PBP6. It was found that the bifunctional carboxy/endopeptidase PBP3 drastically alters the composition of the sacculi with *dacB* mutant sacculi having almost no tripeptide monomers, less tetrapeptide monomers, and more pentapeptide monomers and dimers (Obergfell et al., 2018). Mutation of *dacC* had little effect on sacculi composition. Mutation of *dacB* or *dacC* individually did not affect pilus production, but a double *dacB dacC* mutant had drastically reduced piliation that corresponded with a 94% reduction in transformation efficiency (Obergfell et al., 2018). It is clear that PGases are important for the assembly/stability of Tfp, especially PGases possessing carboxy/endopeptidase activity. The modifications are arguably necessary for allowing the insertion of the large Tfp apparatus with its many associated proteins. Mpg and PBP3 are both D,D-endo/carboxypeptidases. Homologs of DacC are also D,D-endopeptidases, but many Neisseria species have mutations in their three active site motifs (SXXK, SXN, and KTG) all of which are necessary for peptidase activity. *N. meningitidis* DacC only has an intact SXN motif, while *N. gonorrhoeae* DacC lacks all three active site motifs (Obergfell et al., 2018). Perhaps in Neisseria, DacC acts in a complex with PBP3 and directs its activity. Apparently enzymatically functional DacC is present in Gram-negative rods, including the Neisseria species *N. weaveri* and *N. elongata*. The deletion or mutation of multiple genes or gene clusters has been observed in the evolution from a rod to coccoid shape (Veyrier et al., 2015). It is possible that DacC evolved to function as an endopeptidase during elongation but now functions as a scaffold protein to direct Tfp assembly.

Another example of PGases directing the insertion of large protein complexes in the cell wall are those PGases involved with the type IV secretion system (T4SS) that is encoded in the gonococcal genetic island (GGI). The majority of gonococcal strains identified, around 64–80%, have a GGI (Dillard and Seifert, 2001; Pachulec and van der Does, 2010; Wu et al., 2011). The GGI has been identified in 17.5% of *N. meningitidis* strains, and is present in at least two other *Neisseria* spp. (Woodhams et al., 2012; Pachulec et al., 2014; Callaghan et al., 2017). The GGI encodes a T4SS with homology to the *E. coli* F-plasmid in addition to a number of uncharacterized proteins (Callaghan et al., 2017). The Neisseria T4SS functions by secreting single-stranded DNA (Salgado-Pabon et al., 2007). In order for the T4SS to be made and secrete DNA, PGases within the GGI are necessary for the insertion and assembly of the multi-protein secretion system complex into the cell wall. Even though there are multiple LTs and endopeptidases encoded on the chromosome, specific PGases are needed for the T4SS to function. Most GGIs encode two LTs, AtlA and LtgX, both of which are necessary for DNA secretion. Some GGIs have been found to have *eppA*, encoding an M23 endopeptidase, instead of *atlA*. Strains with *eppA* instead of *atlA* are not able to secrete DNA even though other T4SSs, such as that of the F-plasmid, do not require an AtlA homolog (Kohler et al., 2013). It is curious that the T4SS requires multiple LTs while the Tfp requires multiple peptidases for proper assembly and function.

## How Neisseria Differ From the *E. coli* Model

The pathogenic Neisseria differ from the model Gram-negative bacterial species, *E. coli*, in important ways in respect to their PG composition and associated proteins. Some of these differences are due to their respective shapes and the lack in GC of many of the elongation complex proteins. Other differences may be due to differences in classes of proteobacteria. For example, the PG synthesis regulatory proteins, LpoA and LpoB, are only found in γ-proteobacteria (Paradis-Bleau et al., 2010; Typas et al., 2010).

Gonococci and MC are thought to have evolved from rod-shaped bacteria and to have lost the multiple genes encoding parts of the elongation complex (Veyrier et al., 2015). MreB, the filamentous actin-like protein necessary for directing PG synthesis machinery, is absent in GC and MC. Also absent is the *E. coli* PBP2 homolog, a monofunctional D,D-transpeptidase, often associated with MreB that is essential for cell elongation (Zapun et al., 2008). Other MreB-associated proteins absent in GC and MC are the membrane associated proteins MreC, MreD, and RodZ, as well as a glycosyl transferase RodA (Veyrier et al., 2015).

The pathogenic Neisseria lack other PG synthesis proteins or their activators. In *E. coli* the periplasmic outer membrane lipoproteins LpoA and LpoB activate the major bifunctional PG synthases PBP1a and PBP1b, respectively (Typas et al., 2010). GC and MC do not possess LpoA, LpoB, or PBP1b. Lpo homologs are restricted to γ-proteobacteria, which makes the absence of these in β-proteobacteria unsurprising. However, there may be an undiscovered regulator of PBP1 in Neisseria. Interestingly, PBP1, the only bifunctional PBP in Neisseria, is a homolog of PBP1a in *E. coli*. PBP1a is mainly involved with elongation, whereas PBP1b is involved in cell division.

Gram-negative bacteria anchor their outer membrane (OM) to PG. These PG–OM connections have been shown to stabilize the outer membrane and influence the production of outer membrane vesicles. *E. coli* has three known strategies for connecting the OM to PG: one covalent attachment and two types of non-covalent interactions. Braun’s lipoprotein (Lpp) is one of the most abundant proteins in *E. coli*. Lpp is inserted in the inner leaflet of outer membrane at the N-terminus by a lipidated cysteine, and the C-terminus of Lpp is covalently linked to Dap residues in PG by a conserved C-terminal lysine (Braun and Sieglin, 1970; Samsudin et al., 2017). MC and GC lack homologs of Lpp and no other covalent attachments have been reported (Wolf-Watz et al., 1975; Dougherty, 1985; Hill and Judd, 1989). GC and MC also lack the L,D-transpeptidases ErfK, YbiS, YcfS) that are thought to link Lpp to PG (Sanders and Pavelka, 2013). Another related system lacking in GC and MC is the Rcs, or regulator of capsule synthesis system (*rcsBCDF* and *igaA*), that together with Lpp, sense stress by monitoring the size of the periplasmic space (Guo and Sun, 2017; Miller and Salama, 2018).

Non-covalent OM-PG interactions are made in two different ways in *E. coli*, through OmpA and through the Tol-Pal system. The N-terminal domain of OmpA forms an outer membrane porin, while the periplasmic C-terminal domain binds Dap of the macromolecular PG layer. The other interaction is made by a PG-associated lipoprotein, Pal. The N-terminal cysteine of Pal is lipidated and structurally related to the C-terminal PG-binding domain of OmpA (Yamada et al., 1984). Pal is able to further anchor to the inner membrane by binding to the periplasmic TolB that binds to TolA, which is anchored in the inner membrane (Clavel et al., 1998). Neisseria have neither OmpA nor the Tol-Pal system, but they do have two OmpA C-terminal domain containing proteins (OmpA_C-like). One of these OmpA_C-like proteins is NGO1559 and is a predicted outer membrane lipoprotein. Little is known about NGO1559 except that its expression is regulated by iron, and that the protein is found in the outer membrane of GC (Ducey et al., 2005; Zielke et al., 2014).

RmpM (reduction-modifiable protein M) in MC and GC (also called protein III or PIII in GC) defines the other class of OmpA_C-like proteins found in pathogenic Neisseria. RmpM protein was demonstrated to stabilize the outer membrane in MC, specifically through its OmpA_C-like domain (Maharjan et al., 2016). It has been shown to crystalize with outer membrane porin PorB from GC (Zeth et al., 2013). It was also shown to be necessary for localizing the LysM-domain containing protein NGO1873 to the outer membrane, and binds to epithelial cells of the male and female genital tracts (Leuzzi et al., 2013). These proteins could function in alternative pathways to compensate for those absent in Neisseria (e.g., Tol-Pal, Lpp) or they may have other unknown influences on cell wall synthesis, PG remodeling, protein localization, or host attachment.

## *O*-Acetylation of Pg

Modification of PG has been observed in many of the bacteria where PG has been analyzed. *O*-Acetylation of the C6 carbon of MurNAc has been observed in both Gram-positive and Gram-negative bacteria, although the acetylation occurs by different families of proteins (Moynihan and Clarke, 2011). In Gram-negative bacteria, including MC and GC, two acetyltransferase proteins are necessary for PG *O*-acetylation. The first protein is a transmembrane acetyltransferase that functions to transfer an acetyl group past the cytoplasmic membrane to a second acetyltransferase. This second periplasmic acetyltransferase then *O*-acetylates the PG. Both the transmembrane acetyltransferase (PacA) and the periplasmic acetyltransferase (PacB) are necessary for PG *O*-acetylation (Dillard and Hackett, 2005). *O*-Acetylation is known to block host lysozyme from cleaving the PG backbone (Rosenthal, 1979). Another consequence of *O*-acetylation is that it blocks the ability of endogenous LTs to degrade the PG sugar backbone and may thus affect where new PG synthesis occurs.

When *O*-acetylation is blocked by the mutation of *pacA* and/or *pacB* the overall physiology of MC and GC are mostly unchanged. In GC, the absence of PG *O*-acetylation did not affect resistance to human serum, resistance to lysozyme, or PG turnover, but it did increase lysis in the presence of EDTA (Dillard and Hackett, 2005). In MC, the absence of acetylation was shown to have no effect on PG chain length or virulence in a murine sepsis model (Veyrier et al., 2013).

Gram-negative bacteria with *O*-acetylated PG also have an esterase, Ape1, that is able to remove *O*-acetyl groups from PG (Weadge et al., 2005). The ability to de-*O*-acetylate PG is seemingly of greater importance than the ability to *O*-acetylate. In GC, roughly 40% of PG is *O*-acetylated in wild type cells. Deletion of *ape1* did not affect the overall amount of acetylated PG (Dillard and Hackett, 2005). MC cells with an *ape1* deletion had little change in overall *O*-acetylation, but were shown to be significantly larger than wild type or cells with triple *pacA, pacB*, and *ape1* mutations and had longer glycan strands (Veyrier et al., 2013). Virulence in a murine sepsis model was also significantly decreased in an *ape1* single mutant, but not in an *O*-acetylation triple mutant in MC (Veyrier et al., 2013). The same study also showed that Ape1 preferentially acetylates glycans linked to tripeptides (L-Ala-D-Glu-*meso-*Dap). The reason that *apeI* mutants are defective in virulence is not clear, but it could be that the inability to degrade glycan strands leads to this phenotype. In *ltgA ltgD* mutants, which are also defective in degradation of glycan strands, the cells have a defect in envelope integrity and are sensitive to killing by neutrophils and neutrophil-produced elastase and lysozyme (Ragland et al., 2017).

*O*-Acetylation of PG has been observed to be important for a number of other Gram-negative human pathogens such as *Campylobacter jejuni*, *Helicobacter pylori*, and *Proteus mirabilis*, but is not common in many bacteria of a healthy microbiome (Dupont and Clarke, 1991; Wang et al., 2012; Ha et al., 2016). *O*-Acetylation has also been shown to provide lysozyme resistance in the Gram-positive *Enterococcus faecalis*, *Listeria monocytogenes*, *Staphylococcus aureus*, and *Streptococcus pneumoniae* (Moynihan and Clarke, 2011). This process represents a viable option for targeted antimicrobials that do not dramatically alter the microbiome. It also allows for an even more targeted approach by blocking the de-*O-*acetylase of Gram-negative bacteria.

## Pg Recycling

Despite releasing significant amounts of PG fragments into the milieu, *N. gonorrhoeae* and *N. meningitidis* have functional PG recycling systems and recycle a majority of the PG fragments generated during growth. The PG monomers and free disaccharide are taken up into the cytoplasm by the permease AmpG. The amount of PG fragment release vs. PG recycling is one area where these two pathogens have substantial differences in PG metabolism. While *N. gonorrhoeae* releases 15% of the PG monomers generated during growth, *N. meningitidis* only releases 4% of PG monomers (Garcia and Dillard, 2008; Woodhams et al., 2013). That increased PG fragment release is sufficient to increase NOD1 signaling in epithelial cells and the production of IL-8 in human Fallopian tube tissue (Woodhams et al., 2013). The differences in recycling efficiency between *N. gonorrhoeae* and *N. meningitidis* are partly explained by sequence differences in the C-terminal region of AmpG. Three amino acid differences in gonococcal AmpG compared to meningococcal AmpG result in decreased recycling in GC (Chan and Dillard, 2016). The reason these amino acid substitutions affect PG recycling is not clear, but they are not in the region of the protein expected to act in PG fragment binding.

In addition to AmpG, Neisseria also contain the following proteins needed for PG fragment recycling: LdcA, NagZ, AnmK, AmpD, and Mpl. No homolog of MurQ is present, but in other bacteria it has been found that there is an alternative pathway mediated by MupP that converts MurNAc 6-phosphate to MurNAc, bypassing *de novo* synthesis (Borisova et al., 2017) MupP is present in GC (GenBank: EEZ47171.1). Of these enzymes, only AmpD, NagZ, and LdcA have been studied in Neisseria (Garcia and Dillard, 2008; Bhoopalan et al., 2016; Lenz et al., 2017). As mentioned above, LdcA is found as an outer-membrane lipoprotein and thus performs at least some of its functions in the periplasm. However, it was also noted that a smaller soluble form of the protein is produced, and that form of the protein may be in the cytoplasm. Mutation of *ampD* was shown to lead to a build up of MurNAc-peptides in the gonococcal cytoplasm, confirming AmpD’s role in PG recycling (Garcia and Dillard, 2008). NagZ’s role in recycling in GC has not been fully assessed. However, it was shown that purified NagZ was able to remove GlcNAc from PG monomers and free disaccharide. Interestingly, a gonococcal *nagZ* mutant was found to produce thicker biofilms than the wild type, and a moonlighting role was proposed for NagZ in biofilm disassembly as an extracellular glycosidase (Bhoopalan et al., 2016).

Circumstantial evidence suggests that PG breakdown products may be sensed in the gonococcal cytoplasm and their levels may influence PG fragment production or release. We noted that certain PG recycling mutants failed to release free disaccharide even though they were producing free disaccharide in the periplasm. The first example noted was the *ampD* mutant. In this strain, free disaccharide release was nearly abolished. However, an *ampD ampG* double mutant released wild-type levels of free disaccharide, demonstrating that free disaccharide generation in the periplasm was unaffected by the *ampD* mutation (Garcia and Dillard, 2008). Similar results with disaccharide release were obtained with *ltgA* or *ltgD* mutants (Cloud and Dillard, 2002; Cloud-Hansen et al., 2008). Interestingly, PG monomer release was also found to be affected in certain mutants unable to recycle. An *ltgA ltgD* mutant was compared to an *ltgA ltgD ampG* mutant. The *ltgA ltgD* mutant releases little or no PG monomer or free disaccharide, and it is reduced in recycling due to producing less of the anhydro-disaccharide-containing PG fragments recognized by AmpG. When the *ltgA ltgD ampG* mutant was analyzed for fragment release, significant amounts of PG monomers were released indicating that some of this material was generated in the periplasm, but release had somehow been prevented (Schaub et al., 2016). These results suggest that GC are able to regulate PG release and PG uptake into the cytoplasm. Such regulation could be useful for controlling cell wall metabolism or for releasing more or less of the inflammatory PG fragments under different infection conditions.

## Pg and Host Immune Response

The pathology of *N. gonorrhoeae* and *N. meningitidis* infections is due to the host inflammatory response. The bacteria release multiple pro-inflammatory molecules including lipooligosaccharide, porin protein PorB (a TLR2 agonist), heptose-1,7-bisphosphate, and PG fragments (Sinha and Rosenthal, 1980; Kattner et al., 2014; Packiam et al., 2014; Gaudet et al., 2015). The inflammatory response must be advantageous for the bacteria, and for *N. gonorrhoeae*, the ability to attract neutrophils and infect them may be an important step in the disease (Criss et al., 2009). Both NOD1 and NOD2 responses to PG fragments have been observed in the inflammatory responses to *N. gonorrhoeae*. A NOD2 response was described for mice infected with *N. gonorrhoeae*, and a NOD1 response was implicated in human Fallopian tube or epithelial cell studies *in vitro* (Woodhams et al., 2013; Mavrogiorgos et al., 2014).

For humans, NOD1 agonists must contain the second and third amino acids of the PG peptide chain, and the peptide must terminate with DAP (Girardin et al., 2003a; Magalhaes et al., 2005). For GC, the released NOD1 agonists are disaccharide-tripeptide monomer and the free tripeptide (Sinha and Rosenthal, 1980; Chan and Dillard, 2017). Production of the free tripeptide requires AmiC to cleave the peptide from the glycan strand (Lenz et al., 2016). In order for there to be significant amounts of tripeptides in the sacculus, LdcA has to cleave the fourth amino acid (D-Ala) from some of the peptide chains (Lenz et al., 2017). Not surprisingly, AmiC and LdcA were both demonstrated to act in producing NOD1 agonists (Lenz et al., 2016, 2017). However, the requirement for peptidases for NOD1 agonist production was not quite so obvious. Mutations affecting *dacB* (encoding PBP3) and *pbpG* (encoding PBP4) were also demonstrated to decimate NOD1 activation by *N. gonorrhoeae* (Schaub et al., 2019). These enzymes both cleave the common peptide crosslinks (Ala-DAP) and remove the fifth amino acid (D-Ala) (Stefanova et al., 2003; Schaub et al., 2019). When the fifth amino acid is not removed, LdcA cannot cleave the fourth amino acid to leave a strand terminating in DAP (Schaub et al., 2019). Also, the L,D-transpeptidase cannot act to make DAP–DAP crosslinks and in the process cleave an Ala-DAP bond. Thus, no NOD1 agonist is made.

NOD2 is generally described as responding to macromolecular PG such as the whole sacculus or as recognizing muramyl-dipeptide (MurNAc-L-Ala-D-Glu) (Girardin et al., 2003b). *N. gonorrhoeae* and *N. meningitidis* are prone to autolysis, making macromolecular PG available for immune recognition (Bos et al., 2005; Chan et al., 2012). Furthermore, large PG fragments have been demonstrated to induce pathology in a rat model of gonococcal arthritis (Fleming et al., 1986). *O*-Acetylation of PG decreases its destruction by lysozyme and may explain its greater effects on arthritis (Fleming et al., 1986; Dillard and Hackett, 2005). *N. gonorrhoeae* and *N. meningitidis* do not release muramyl dipeptide (Sinha and Rosenthal, 1980). However, they do release a soluble PG molecule that is converted by the host into a NOD2 agonist. Glycosidically linked dimers are released by both pathogens, and when these molecules are digested by host lysozyme, the PG monomers carrying a reducing end on MurNAc are potent agonists for NOD2 (Woodhams et al., 2013; Dagil et al., 2016; Knilans et al., 2017). When PG dimers are degraded by the bacterial glycosidases, such as LtgA or LtgD, the products all have a 1,6-anhydro bond on the MurNAc and do not stimulate NOD2 (Knilans et al., 2017). It is interesting to note that commensal Neisseria species *N. sicca* and *N. mucosa* do not produce PG dimers, suggesting that the lack of a NOD2 response may be helpful for these bacteria to maintain a non-pathogenic lifestyle (Chan and Dillard, 2016).

We recently made the observation that one common *N. gonorrhoeae* strain induces an unusually high NOD2 response. Strain FA19 was found to cause a large NOD2 response in epithelial cells both to soluble PG fragments released by the bacteria during growth and to purified sacculi. Compositional analyses demonstrated that both the sacculus and the released fragments contained much larger amounts of dipeptide chains compared to other gonococcal strains (Schaub et al., 2019). This observation suggests that Neisseria have another, yet uncharacterized, endopeptidase and that this enzyme may differ between strains or may be regulated to give larger or smaller NOD2 responses.

## Concluding Remarks

All *N. gonorrhoeae* strains analyzed to date have a somewhat defective version of the PG fragment permease AmpG, making it likely that all GC release a substantial amount of their PG fragments generated during growth (Chan and Dillard, 2016). Understanding the roles of these PG fragments in infections and the host responses to them will continue to progress as more sophisticated infection models are developed and as we learn more from *ex vivo* human organ culture models. Even without considering effects on infection, the Neisseria make an attractive model for understanding PG metabolism generally. With the ability to do natural transformation and the small number of PG metabolism genes, the Neisseria make a promising system for revealing the functions of PG degradation and synthesis proteins. *N. gonorrhoeae* and *N. meningitidis* are coccal in shape, but they have close relatives that are rod shaped including *N. elongata* and *N. bacilliformis*. Thus the evolution and advantages of coccal shape might be further explored as has begun with the studies by Veyrier et al. (2015). The tendency of the bacteria to undergo autolysis adds another area of interest for understanding phenomena related to infection and inflammation as well as release of DNA for natural transformation (Hebeler and Young, 1975; Hamilton and Dillard, 2006). Key areas for future investigation involve understanding protein–protein interactions and mechanisms of regulation and sensing of PG synthesis and degradation. The AmpR-based method of PG sensing in the cytoplasm is not present in many bacterial species and is not present in Neisseria (Jacobs et al., 1994; Chan and Dillard, 2017). However, there is evidence of PG fragment sensing, suggesting an unexplored mechanism exists (Cloud-Hansen et al., 2008; Garcia and Dillard, 2008). As mentioned above, the LpoA–LpoB mechanism of regulating PG biosynthesis is also absent in many bacterial species, so an unknown mechanism is likely to be identified for that process as well. Protein–protein interactions for Neisseria PG metabolism proteins have been found as in other bacteria, and it is likely that further investigation of this simplified system will reveal more information about coordinated activities, enzyme activation, and regulation (Lenz et al., 2016; Chan and Dillard, 2017; Perez Medina and Dillard, 2018).

## Author Contributions

This review was written with contributions from RS and JD.

## Conflict of Interest Statement

The authors declare that the research was conducted in the absence of any commercial or financial relationships that could be construed as a potential conflict of interest.
